# Maize Plant Morphology Affects Resistance to Stalk Breaking by Affecting Plant Stress

**DOI:** 10.3390/plants14111598

**Published:** 2025-05-24

**Authors:** Yujie Cao, Ming Tian, Shang Gao, Bo Ming, Keru Wang, Haibing Yu, Shaokun Li, Jun Xue

**Affiliations:** 1 School of Agriculture, Anhui Science and Technology University, Chuzhou 233100, China; yujiecao99@163.com (Y.C.); hsm50721@163.com (H.Y.); 2 Institute of Crop Sciences, Chinese Academy of Agricultural Sciences/Key Laboratory of Crop Physiology and Ecology, Ministry of Agriculture and Rural Affairs, Beijing 100081, China; 18748143885@163.com (M.T.); g382824817@126.com (S.G.); mingbo@caas.cn (B.M.); wangkeru@caas.cn (K.W.)

**Keywords:** maize, torque, leaf area, fresh weight, center of gravity height, critical wind speed of stalk-breaking, wind tunnel test

## Abstract

The critical wind speed for stalk breaking is a direct and rapid method for evaluating stalk-breaking resistance. Maize lodging resistance is determined by the plant’s wind-induced stress and the stalk’s mechanical strength, yet the factors influencing plant stress remain unclear. This study analyzed the quantitative relationship between plant leaf area, weight, and stalk base torque by implementing different leaf-cutting and ear-removal treatments. The key factors affecting plant stress under varying wind speed conditions were identified. Results indicated that the critical wind speed for stalk breaking significantly increased following leaf cutting and ear removal. Under different wind speed conditions, stalk base torque exhibited a significantly negative correlation with the critical wind speed for stalk breaking, with the strongest correlation observed at U = 14.6 m s^−1^. At this wind speed, every 1 m^2^ increase in leaf area resulted in a torque increase of 6.7 N m and a decrease in critical wind speed for stalk breaking by 17.5 m s^−1^. Similarly, every 1 kg increase in plant fresh weight led to an 8.1 N m torque increase and an 18.3 m s^−1^ decrease in critical wind speed. Additionally, every 1 m increase in the height of the center of gravity resulted in a torque increase of 13.3 N m and a 22.9 m s^−1^ reduction in critical wind speed. Regression analysis revealed that changes in critical wind speed for stalk breaking were primarily influenced by leaf area and plant fresh weight, which accounted for 80.6% of its variation. The effects of plant fresh weight and leaf area on torque varied under different wind speed conditions. In conclusion, maize leaf area, fresh weight, and center of gravity height influence the critical wind speed for stalk breaking by altering plant torque in a wind environment.

## 1. Introduction

Maize is an important food crop and feed crop, ranking highest in global and Chinese planting areas and total yield [1]. As a tall, gramineous crop, maize is prone to stalk lodging or fracture due to meteorological factors such as wind and rain [2]. Lodging significantly reduces crop yield and grain quality while lowering mechanical harvesting efficiency [3,4]. Severe lodging can cause a 30% to 50% reduction in yield, with China experiencing annual maize yield losses of nearly one million tons. The more serious the lodging degree, the greater the loss of maize yield [5,6].

The primary causes of maize root lodging and stalk breakage are mechanical instability, external forces such as wind, or a combination of both [7]. Root lodging mainly occurs between the six-leaf expansion and silking stages while the plant is still growing [8,9]. Partial recovery is possible through root stabilization or manual straightening. In contrast, stalk breaking typically occurs from the rapid elongation stage (nine-leaf expansion) to harvest [10]. Broken stalks disrupt the plant’s conduction tissue, leading to insufficient water and nutrient supply, resulting in greater yield loss [8,11,12]. Understanding the effects of wind on plant lodging is essential for revealing the dynamic mechanisms of wind-induced stalk breakage and providing a scientific basis for breeding lodging-resistant varieties, optimizing cultivation practices, and developing harvesting strategies. This research is critical for ensuring maize yield, improving mechanized harvesting efficiency, and addressing climate change challenges.

Current maize lodging resistance evaluation methods are categorized as direct or indirect. Accurate assessment of stalk lodging resistance supports breeding programs and cultivation regulation technologies. Direct evaluation involves field lodging rate assessments [13,14], but these are highly dependent on external wind conditions. Indirect methods on mechanical and material engineering indices include stalk breaking force, pushing force [15,16,17,18], rind penetration strength [19,20,21], crushing strength [22,23], and bending strength [24,25,26,27]. Additional parameters include geometric characteristics (cross-sectional inertia moment) and material properties (elastic modulus, bending stiffness) [27,28,29]. While these indices reflect stalk strength, their correlation with lodging resistance remains unclear, necessitating further research. Some researchers have introduced lodging resistance indices for comprehensive evaluation [30,31], but the weight distribution of different methods remains controversial.

Since maize lodging results from external wind conditions, existing evaluation methods only partially reflect lodging resistance. Despite their accuracy, they do not account for plant stress states or fully represent field lodging scenarios. Wind tunnel testing bridges this gap by simulating field conditions and assessing plant stress under various wind speeds, offering high efficiency and low cost for maize lodging resistance evaluation [32,33,34,35]. However, laboratory-based tests often fail to replicate natural field conditions, leading to discrepancies in plant growth and development. Additionally, indoor planting incurs high costs, slow measurement speeds, and limitations on large-scale sample testing.

Stalk bending occurs from wind forces and plant gravity [36], while stalk strength depends on its geometry and material properties [37]. When the bending moment exceeds the stalk’s capacity, breakage occurs [38]. Factors influencing stalk bending moments include plant morphology traits such as aboveground fresh weight, ear height, center of gravity height, and leaf angle [36]. Studies suggest that reducing the windward area by cutting leaves and lowering plant height can enhance lodging resistance [39,40,41,42]. Varieties with high ear positions have weaker lodging resistance due to higher centers of gravity, whereas low-ear varieties exhibit stronger lodging resistance. Dwarf maize varieties are widely used in production [42], and chemical control methods have been employed to reduce ear height and improve lodging resistance [42,43].

Stalk strength is determined by stalk morphology, anatomical structure, cell wall composition, and cellular turgor. The cross-sectional shape of internodes is closely related to lodging resistance, with thicker internodes offering greater bending resistance [27,28]. Lignin content also plays a crucial role in stalk bending resistance [44], and maize varieties with thick outer tissues exhibit stronger resistance to breakage [45,46]. While previous studies have extensively examined maize stalk fracture factors, most studies focus solely on the plant’s inherent stress capacity, leaving gaps in understanding the relationship between plant morphology, stress, and stalk fracture resistance.

A correlation exists between critical wind speed and lodging rate [7]. Since differences in year, cultivar, and treatment will change the stress resistance of maize plants, which in turn affects the lodging resistance of maize, this study investigated two maize hybrids widely planted in China, Zhengdan 958 and Xianyu 335, in 2019 and 2024. By modifying leaf area, weight, and center of gravity height through leaf cutting and ear removal, we measured plant base torque under different wind speeds. Our study established a quantitative relationship between leaf area, weight, center of gravity height, and maize torque under varying wind conditions. Additionally, a quantitative model linking critical wind speed to stalk fracture factors was developed. These findings provide a theoretical foundation for breeding lodging-resistant maize varieties and refining cultivation and regulation strategies.

## 2. Results

### 2.1. Critical Wind Speed of Stalk Breaking

**Figure 1:**
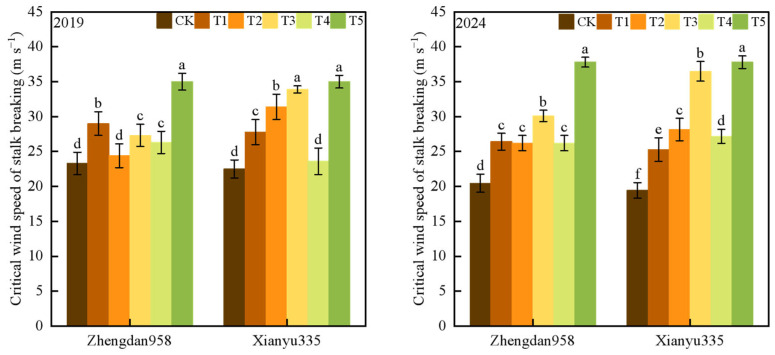
The difference in critical wind speed of stalk breaking between different treatments. Note: CK—control group (no treatment), T1—removal of all leaves below the ear leaf, T2—removal of all leaves above the ear leaf, T3—removal of all leaves, T4—removal of the female ear while retaining all leaves, T5—removal of all leaves and the ear. Different lowercase letters indicate significant differences at the 0.05 level between different treatments of the same variety in the same year.

Compared with CK, the critical wind speed of stalk breaking increased significantly after leaf cutting and ear removal (Figure 1). Compared with the no treatment group, the average critical wind speed of stalk breaking in removal of all leaves below the ear leaf treatment (T1) and above the ear leaf area treatment (T2) increased by 26.8% and 29.3%, respectively, and the average critical wind speed of stalk breaking in removal of all leaves treatment (T3), removal of the female ear while retaining all leaves (T4), and removal of all leaves and the ear treatment (T5) increased by 50.7%, 21.4%, and 71.2%, respectively. These results indicate that removing all leaves had a greater effect on the critical wind speed of maize stalk breaking than removing the ear. Compared with the treatment of removing the lower leaves of the ear, removing the upper leaves had a more significant impact on the critical wind speed of stalk breaking.

### 2.2. Torque

**Figure 2:**
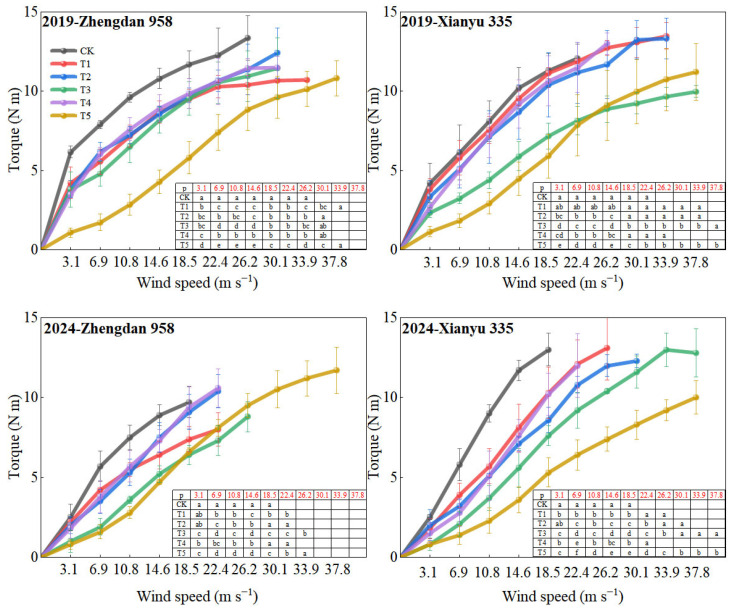
Changes in plant torque with wind speed under different treatments. Note: CK—control group (no treatment), T1—removal of all leaves below the ear leaf, T2—removal of all leaves above the ear leaf, T3—removal of all leaves, T4—removal of the female ear while retaining all leaves, T5—removal of all leaves and the ear. Different lowercase letters indicate significant differences at the 0.05 level between different treatments under the same wind speed conditions.

AS wind speed increased, the torque of maize plants also increased, with the difference between treatments becoming more pronounced (Figure 2). Under the same wind speed conditions, the torque at the base of the CK treatment was the highest, while the lowest torque was observed in the ear removal + leaf cutting treatment (T5). Over the two years, the torque performance of Xianyu 335 followed the order: CK > lower ear leaf removal (T1) ≥ ear leaf removal (T4) > upper ear leaf removal (T3) > all leaf removal (T3) > ear leaf cutting (T5). Similarly, for Zhengdan 958, the torque changes among treatments followed the order: CK > ear removal (T4) ≥ ear removal upper leaf (T2) > ear removal lower leaf (T1) > all leaf removal (T3) > ear removal leaf cutting (T5). Within a wind speed range of 3.1 m s^−1^ to 22.4 m s^−1^, the torque of the lower leaf removal treatment and the upper leaf removal treatment decreased by 20.3%, 23.4%, 24%, 21.2%, 16.1%, 8.9% and 24.6%, 30.4%, 27.4%, 22.6%, 16.2%, 10.3%, respectively, compared with CK.

Correlation analysis showed that when the wind speed ranged from 3.1 m s^−1^ to 30.1 m s^−1^, the torque at the maize stalk base was significantly negatively correlated with the critical wind speed of stalk breaking (Table 1). When *n* = 24, the highest correlation coefficient between torque and critical wind speed of stalk breaking was observed at a wind speed (U) of 14.6 m s^−1^.

### 2.3. Leaf Area

**Figure 3:**
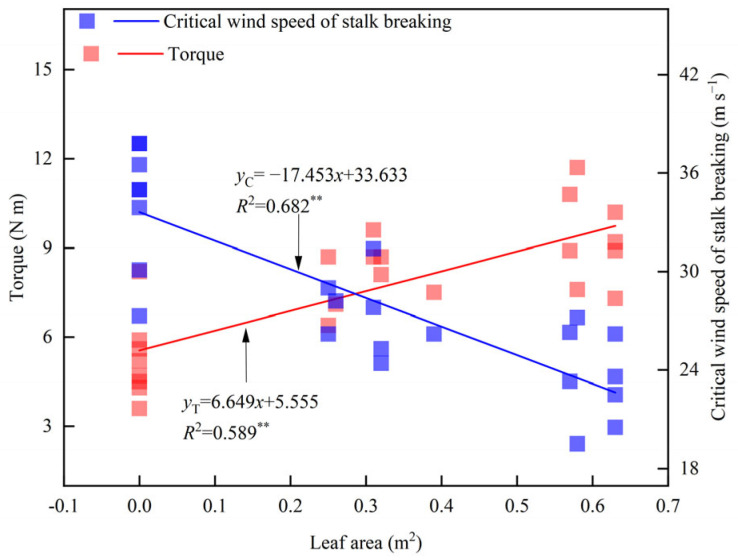
Relationship between leaf area and plant torque (U = 14.6 m s^−1^) and critical wind speed of stalk breaking. ** indicates significance at 0.01.

The leaf area per plant decreased significantly after leaf cutting (Table 2). Compared with CK, the leaf area was reduced by an average of 53% in T1 and 47% in T2. The T4 treatment had no effect on the leaf area. Linear fitting analysis indicated that leaf area was significantly negatively correlated with the critical wind speed of stalk breaking and significantly positively correlated with torque at a wind speed of 14.6 m s^−1^ (Figure 3). For every 1 m^2^ increase in leaf area, torque increased by 6.6 N m, while the critical wind speed of stalk breaking decreased by 17.5 m s^−1^.

### 2.4. Plant Fresh Weight

**Figure 4:**
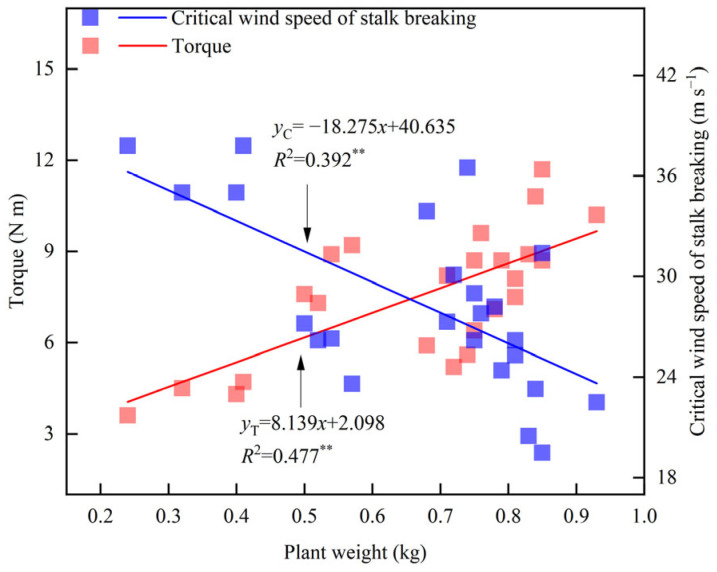
Relationship between fresh weight and torque (U = 14.6 m s^−1^) and critical wind speed. ** indicates significance at 0.01.

Leaf cutting and ear removal significantly affected the fresh weight of maize (Table 3). Compared with CK, the fresh weight of maize decreased by 10.8%, 6.2%, and 17.1% after removing the lower ear leaf (T1), the upper ear leaf (T2), and the whole ear leaf (T3), respectively. The fresh weight of maize decreased by 38.2% after removing the female ear (T4), and by 60.1% after cutting the leaf and removing the ear (T5). The fresh weight of Zhengdan 958 followed the order CK > T2 > T1 > T3 > T4 > T5. In 2019, the fresh weight of Xianyu 335 followed the same order, whereas in 2024, it followed CK > T1 > T2 > T3 > T4 > T5. Linear fitting analysis showed that plant fresh weight was significantly negatively correlated with the critical wind speed of stalk breaking and significantly positively correlated with torque at a wind speed of 14.6 m s^−1^ (Figure 4). For every 1 kg increase in plant fresh weight, the torque increased by 8.1 N m, while the critical wind speed of stalk breaking decreased by 18.3 m s^−1^.

### 2.5. Height of Center of Gravity

**Figure 5:**
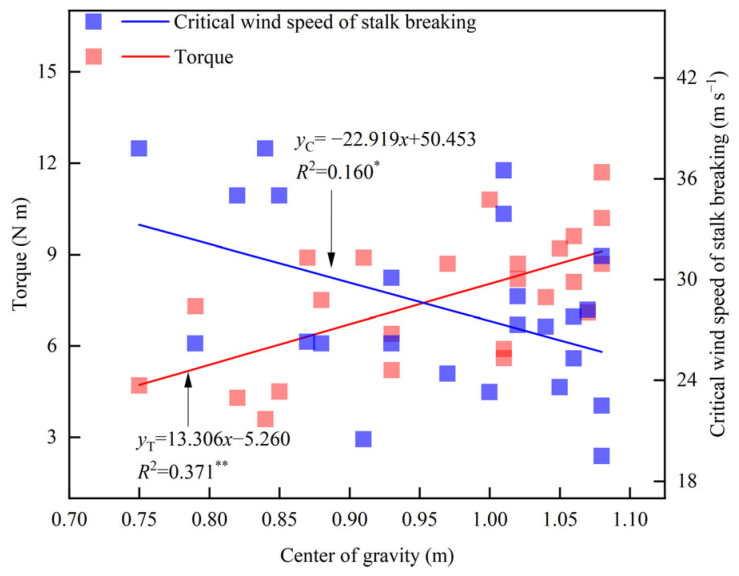
Relationship between the height of the center of gravity, plant torque (U = 14.6 m s^−1^), and critical wind speed of stalk breaking. * indicates significance at 0.05, ** indicates significance at 0.01.

Leaf cutting and ear removal treatments significantly affected the height of the center of gravity of maize (Table 4). Compared with the CK of Zhengdan 958, the height of the center of gravity increased by 2.1% in the lower leaf removal treatment (T1) and in the whole leaf removal treatment (T3). Conversely, the height of the center of gravity decreased by 3.2% in the upper leaf removal treatment (T2), by 13.1% in the ear removal treatment (T4), and by 17.8% in the leaf-cutting and ear removal treatment (T5). In Xianyu335, the height of the center of gravity decreased by 1.9% (T1), 0.4% (T2), 6.5% (T3), 3.2% (T4), and 21.8% (T5) on average compared with that of CK. Linear fitting analysis showed that the height of the center of gravity was significantly negatively correlated with the critical wind speed of stalk breaking and significantly positively correlated with torque at a wind speed of 14.6 m s^−1^ (Figure 5). For every 1 m increase in the height of the center of gravity, the torque increased by 13.3 N m, while the critical wind speed of the stalk break decreased by 22.9 m s^−1^.

### 2.6. Factors Affecting the Critical Wind Speed of Maize Stalk Breakage and Torque

Stepwise regression analysis showed that the relationship between critical wind speed (*y*) and leaf area (*x*_1_) and fresh weight (*x*_2_) was y = −14.386 *x*_1_ − 10.892 *x*_2_ + 40.004, R^2^ = 0.806 **, *n* = 24, indicating that leaf area and fresh weight accounted for 80.6% of the variation in critical wind speed.

**Figure 6:**
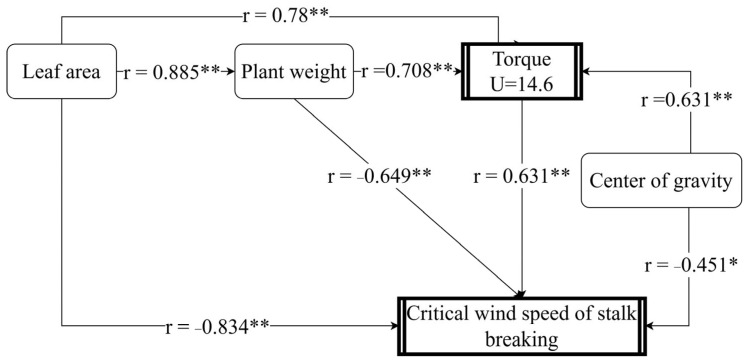
Path analysis of critical wind speed, torque, center of gravity height, leaf area, and fresh weight of maize plants. * indicates significance at 0.05, ** indicates significance at 0.01.

The path analysis model was further established to assess the influence of plant torque on the critical wind speed of maize stalk breakage (Figure 6). Leaf area has a direct effect on the critical wind speed for stalk breaking, and there is a significant positive correlation between the size of the leaf area and the fresh weight of the plant. Additionally, leaf area has an indirect effect on the critical wind speed for stalk breakage through fresh weight and torque. The results indicated that leaf area had the highest direct impact on the critical wind speed of stalk breaking, followed by plant fresh weight, while the height of the center of gravity had the lowest direct impact. Leaf area, plant fresh weight, and height of the center of gravity had significant direct effects on plant torque at a wind speed of 14.6 m s^−1^ (*p* ≤ 0.01) and indirect effects on the critical wind speed of stalk breaking. These findings suggest that leaf area, plant fresh weight, and center of gravity height influence the critical wind speed of stalk breaking by affecting plant torque.

Key indicators affecting plant torque varied under different wind speed conditions. Stepwise regression analysis showed that plant torque was primarily determined by fresh weight at wind speeds of 3.1 m s^−1^ and 33.9 m s^−1^ (Table 5). At a wind speed of 30.1 m s^−1^, plant torque was mainly determined by leaf area. At wind speeds of 6.9–14.6 m s^−1^, plant torque was predominantly determined by plant fresh weight and leaf area, explaining 61.0% to 78.5% of the variation in torque. Under moderate wind speeds of 18.5–26.2 m s^−1^, plant torque was primarily influenced by leaf area and the center of gravity height, which explained 75.5% to 81.9% of the change in torque.

## 3. Discussion

Wind is the most significant environmental factor causing maize lodging, and the critical wind speed for stalk breakage serves as a comprehensive index to evaluate maize wind resistance [47,48,49,50]. Studies have shown that the critical wind speed from stalk breakage is significantly negatively correlated with the natural lodging rate in the field [7]. Lodging resistance was negatively correlated with plant weight, leaf area, and center of gravity, while the lodging rate was significantly positively correlated with the center of gravity [51,52,53,54,55,56]. Maintaining a lower center of gravity height can more effectively reduce lodging [14]. The findings of predecessors demonstrated that maize lodging was positively correlated with wind speed [57]. Therefore, plant weight, center of gravity, and leaf area are important factors for evaluating lodging resistance. Analysis in this study revealed that the critical wind speed for stalk breakage was significantly negatively correlated with plant weight and leaf area, and significantly negatively correlated with the center of gravity height. This study differs from previous studies, reinforcing that the critical wind speed for stalk breakage is an important index of maize lodging resistance when combined with plant weight, leaf area, and center of gravity.

Changes in torque at the base of the maize plant were used to measure the plant’s stress state under different wind speeds. Torque is determined by force and the action point of the force [58,59]. Lodging force is determined by wind and the aerodynamic resistance of the upper canopy and is also related to planting density, leaf area, and leaf angle [60]. Plant lodging is closely related to the morphology of maize and other major crops [61,62]. Lodging occurs when lodging torque exceeds lodging resistance [38]. The results of this study showed that torque increased with wind speed until the stalk broke for the same variety. There were always torque differences between different treatments of the same variety. In cases where the stalk did not break at the same external wind speed, the torque differed among varieties. This may be because wind conditions are influenced by multiple factors, including plant fresh weight, leaf area, and center of gravity height. Correlation analysis showed a significant positive correlation between torque and fresh weight, leaf area, and center of gravity, with leaf area having the greatest influence on torque. This was because leaf area size, center of gravity position, and weight affect the plant’s windward area and bending angle under external force, determining the size of the wind arm and gravity arm. Physical modifications to leaf area and plant weight altered the center of gravity and windward area of the plant. This study verified the hypothesis proposed by previous researchers that lodging is related to plant morphology and expanded upon their findings by deriving a fitting formula for different plant morphologies under varying wind speed conditions.

The upper leaf area is the primary part affected by wind. Previous studies have shown that the wind torque is directly affected by vertical leaf area distribution and wind speed, with the vertical leaf area distribution determining wind torque at a given wind speed [63]. Some studies have shown that removing upper leaves at the flowering stage can increase carbohydrate content, puncture strength, and stalk lodging resistance [53]. A smaller top leaf area reduces wind torque on plants [64]. Computational Fluid Dynamics (CFD) model studies of regular cubes indicate the existence of compensatory airflow on one side of the canopy, which enters from the upper part and exits from the lower part [65]. Field studies have found that most lodging occurs in the middle of the field [33]. All the above studies have demonstrated the significance of the upper leaf area of maize plants in resisting lodging. The results of this study show that compared with the no treatment group, the average critical wind speed of stalk breaking in the removal of all leaves below the ear leaf treatment (T1) and above the ear leaf area treatment (T2) increased by 26.8% and 29.3%, respectively. Within a wind speed range of 3.1 m s^−1^ to 22.4 m s^−1^, the torque of the lower leaf removal treatment and the upper leaf removal treatment decreased by 20.3%, 23.4%, 24%, 21.2%, 16.1%, 8.9% and 24,6%, 30.4%, 27.4%, 22.6%, 16.2%, and 10.3%, respectively, compared with CK. Compared with CK, the torque drop of T1 is smaller than that of T2 under the same wind speed. This indicates that the upper leaf area has a greater impact on torque than the lower leaf area. This might be due to the use of a turbofan in this study, where the wind pressure at the upper part of its outlet is greater than that at the lower part [33]. Therefore, upper leaves contribute more to the torque of maize plants and the critical wind speed for stalk breakage. This finding is consistent with previous studies, which have shown that airflow above the canopy is significant [66,67]. Therefore, in the future, the resistance of maize to stalk breakage can be enhanced by molecular breeding research and spraying plant growth regulators to adjust the upper leaf area of maize ear leaves.

## 4. Materials and Methods

### 4.1. Experimental Treatment Design

The experiment was conducted at the Xinxiang Comprehensive Experimental Station of the Chinese Academy of Agricultural Sciences during the summer maize growing seasons of June to October in 2019 and 2024. Zhengdan 958 and Xianyu 335 were used as experimental materials. The planting density was set at 75,000 plants ha, with an equal row spacing of 0.6 m. Field management was carried out in accordance with local agricultural practices.

**Figure 7:**
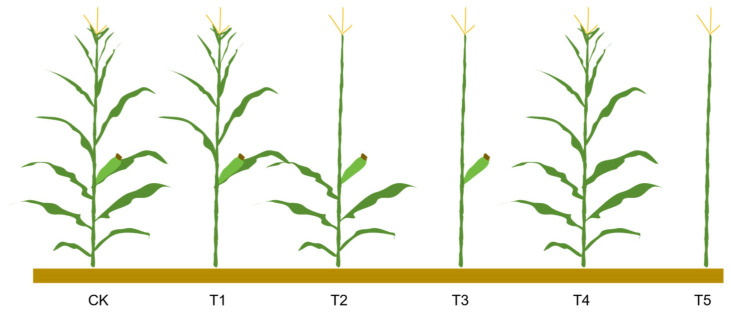
Schematic diagram of experimental treatments. Note: CK, T1, T2, T3, T4, and T5 represent the control group (no treatment), removal of all leaves below the ear leaf, removal of all leaves above the ear leaf, removal of all leaves, removal of the female ear while retaining all leaves, and removal of all leaves and the ear, respectively.

At 20 days after silking, representative plants exhibiting uniform growth were randomly selected in the field. Five experimental treatments were established (Figure 7): (T1) removal of all leaves below the ear leaf, (T2) removal of all leaves above the ear leaf, (T3) removal of all leaves, (T4) removal of the female ear while retaining all leaves, and (T5) removal of all leaves and the ear. Plants in their natural field state served as the control (CK). The critical wind speed, plant torque, plant leaf area, plant weight, and center of gravity height were measured for each treatment, with 5 replicates per treatment.

### 4.2. Measurement and Methods

Maize stalk lodging follows the “minimum resistance law”. Therefore, when measuring wind speed and torque, the long axis of the stalk was positioned perpendicular to the air outlet [68,69,70].

(1) Height of the center of gravity: The plant was placed horizontally (with spikes, leaves, and sheaths intact), and balance was achieved by lifting it with an index finger. The distance from the balancing point to the base of the stalk was recorded as the height of the center of gravity [10,55].
(2) Fresh weight of the plant: Following plant morphology measurements, the fresh weight of the whole plant was recorded. At the conclusion of the fan test, the ear leaf sheath was removed to fully expose the ear, and the fresh weight of the female ear was measured after complete removal.
(3) Leaf area: The length (L) and width (W) of the longest and widest portions of each leaf were measured using a ruler. The total leaf area of the plant was determined by summing the areas of all of the leaves. The single leaf area was calculated using the formula:

Single leaf area = (L) × (W) × 0.75

**Figure 8:**
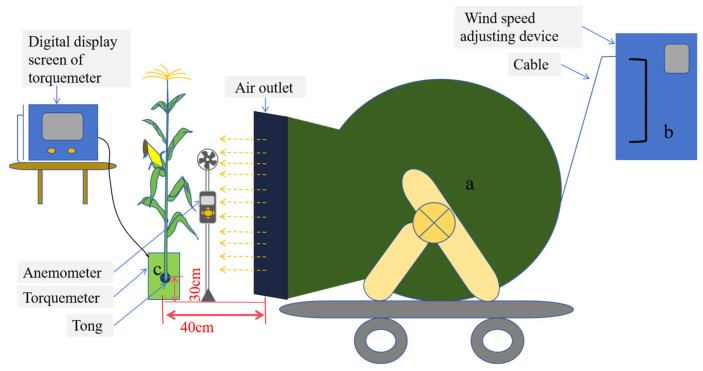
Measurement diagram of critical wind speed and base torque of maize stalk breakage. a—a support structure and an electric turbofan, b—an inverter, and c—a fixed structure and torque meter.

(4) Critical wind speed of stalk breaking: A self-developed crop lodging resistance measurement device was used to determine the critical wind speed (Figure 8). The plant was cut at the base and fixed vertically on a bracket at the first internode above ground level. The plant was positioned 40 cm away from the air outlet, with the base of the plant set 30 cm above the outlet. The wind speed was increased gradually at a constant rate, with ten wind speed levels set based on motor frequency (0–50 Hz): 3.1 m s^−1^, 6.9 m s^−1^, 10.8 m s^−1^, 14.6 m s^−1^, 18.5 m s^−1^, 22.4 m s^−1^, 26.2 m s^−1^, 30.1 m s^−1^, 33.9 m s^−1^, and 37.8 m s^−1^. The wind speed at which the plant bent and subsequently broke was recorded as the critical wind speed of stalk breaking.
(5) Plant torque: After measuring the critical wind speed of stalk breaking, plant torque under different wind speed conditions was determined using a torque meter (Figure 8c). The torque measured in this study was generated by external forces, including wind and gravity. Torque will no longer be recorded when the stalk breaks.

### 4.3. Data Analysis

Data processing was performed using Microsoft Excel 2010. Statistical analysis was conducted using SPSS 23, and figures were generated with Origin 2019. The least significant difference (LSD) test was applied to compare differences between treatments (*p* < 0.05). Pearson correlation analysis was used to evaluate the relationship between torque and the critical wind speed of stalk breaking. Path analysis was employed to assess relationships between leaf area, plant fresh weight, center of gravity height, torque, and critical wind speed of stalk breaking. Stepwise regression analysis was used to identify key indicators affecting torque and critical wind speed under varying wind speed conditions.

## 5. Conclusions

After leaf-cutting and ear-removal treatments, the critical wind speed of stalk breaking increased, the basal torque decreased, the leaf area and plant fresh weight decreased, and the center of gravity height increased and decreased. When the wind speed is less than 33.9 m s^−1^, the torque is significantly negatively correlated with the critical wind speed of stalk breakage, and the torque at the base of the stalk of maize reflects the stress state of the plant under the wind environment, and the torque can be used as an important index to evaluate the resistance of maize to stalk bending, and torque increases with the increase of wind speed. Leaf area, fresh weight, and center height of maize were significantly negatively correlated with the critical wind speed of stalk folding, and positively correlated with plant torque. Wind affects the stress state of plants through leaf area, fresh weight, and center of gravity height, which in turn affects the lodging resistance of plants, and the key factors affecting the critical wind speed of stalk break are different under different wind speeds.

## Figures and Tables

**Table 1:** Correlation between torque and critical wind speed of stalk breaking at different wind speeds.

	Critical Wind Speed of Stalk Breaking
Torque_U=3.1_ (*n* = 24)	−0.577 **
Torque_U=6.9_ (*n* = 24)	−0.783 **
Torque_U=10.8_ (*n* = 24)	−0.847 **
Torque_U=14.6_ (*n* = 24)	−0.86 **
Torque_U=18.5_ (*n* = 24)	−0.821 **
Torque_U=22.4_ (*n* = 24)	−0.713 **
Torque_U=26.2_ (*n* = 21)	−0.79 **
Torque_U=30.1_ (*n* = 19)	−0.589 **
Torque_U=33.9_ (*n* = 14)	−0.419 ns
Torque_U=37.8_ (*n* = 6)	−0.286 ns

Note: U indicates wind speed, *n* indicates the sample size, ** indicates significance at the 0.01 probability level, and ns indicates no significant correlation.

**Table 2:** Differences in maize plant leaf area between different treatments.

Variety	Treatment	Leaf Area (m^2^)	
		2019	2024
Zhengdan 958	CK	0.57 ± 0.06 a	0.63 ± 0.04 a
	T1	0.25 ± 0.02 c	0.25 ± 0.03 c
	T2	0.32 ± 0.05 b	0.39 ± 0.03 b
	T3	0	0
	T4	0.57 ± 0.06 a	0.63 ± 0.04 a
	T5	0	0
Xianyu 335	CK	0.63 ± 0.03 a	0.58 ± 0.06 a
	T1	0.31 ± 0.04 b	0.32 ± 0.03 b
	T2	0.31 ± 0.02 b	0.26 ± 0.04 c
	T3	0	0
	T4	0.63 ± 0.03 a	0.58 ± 0.06 a
	T5	0	0

Note: CK—control group (no treatment), T1—removal of all leaves below the ear leaf, T2—removal of all leaves above the ear leaf, T3—removal of all leaves, T4—removal of the female ear while retaining all leaves, T5—removal of all leaves and the ear. Different lowercase letters indicate significant differences at the 0.05 level between different treatments of the same variety in the same year.

**Table 3:** Differences in fresh weight of maize plants across treatments.

Variety	Treatment	Plant Fresh Weight (kg Plant ^−1^)	
		2019	2024
Zhengdan 958	CK	0.84 ± 0.07 a	0.83 ± 0.09 a
	T1	0.75 ± 0.03 b	0.75 ± 0.06 b
	T2	0.79 ± 0.08 b	0.81 ± 0.07 a
	T3	0.71 ± 0.06 c	0.72 ± 0.07 b
	T4	0.54 ± 0.05 d	0.52 ± 0.04 c
	T5	0.40 ± 0.01 e	0.41 ± 0.06 d
Xianyu 335	CK	0.93 ± 0.05 a	0.85 ± 0.07 a
	T1	0.76 ± 0.09 c	0.81 ± 0.02 ab
	T2	0.85 ± 0.05 b	0.78 ± 0.06 bc
	T3	0.68 ± 0.08 d	0.74 ± 0.08 c
	T4	0.57 ± 0.03 e	0.50 ± 0.06 d
	T5	0.32 ± 0.02 f	0.24 ± 0.03 e

Note: CK—control group (no treatment), T1—removal of all leaves below the ear leaf, T2—removal of all leaves above the ear leaf, T3—removal of all leaves, T4—removal of the female ear while retaining all leaves, T5—removal of all leaves and the ear. Different lowercase letters indicate significant differences at the 0.05 level between different treatments of the same variety in the same year.

**Table 4:** Differences in the height of the center of gravity in maize plants across treatments.

Variety	Treatment	Center of Gravity (m)	
		2019	2024
Zhengdan 958	CK	1.00 ± 0.002 c	0.91 ± 0.003 b
	T1	1.02 ± 0.001 b	0.93 ± 0.008 a
	T2	0.97 ± 0.002 d	0.88 ± 0.002 c
	T3	1.02 ± 0.003 a	0.93 ± 0.002 a
	T4	0.87 ± 0.001 e	0.79 ± 0.002 d
	T5	0.82 ± 0.005 f	0.75 ± 0.006 e
Xianyu 335	CK	1.08 ± 0.004 a	1.08 ± 0.005 a
	T1	1.06 ± 0.002 c	1.06 ± 0.005 c
	T2	1.08 ± 0.001 b	1.07 ± 0.002 b
	T3	1.01 ± 0.001 e	1.01 ± 0.004 e
	T4	1.05 ± 0.005 d	1.04 ± 0.001 d
	T5	0.85 ± 0.005 f	0.84 ± 0.001 f

Note: CK—control group (no treatment), T1—removal of all leaves below the ear leaf, T2—removal of all leaves above the ear leaf, T3—removal of all leaves, T4—removal of the female ear while retaining all leaves, T5—removal of all leaves and the ear. Different lowercase letters indicate significant differences at the 0.05 level between different treatments of the same variety in the same year.

**Table 5:** Regression analysis of plant torque in relation to fresh weight, leaf area, and center of gravity height under different wind speed conditions.

Wind Speed (m s^−1^)	Regression Equation	R^2^	*n*
U = 3.1	y = 4.211 *x*_2_ − 0.341	0.307 *	24
U = 6.9	y = 3.622 *x*_1_ + 4.533 *x*_2_ + 0.012	0.61 **	24
U = 10.8	y = 4.681 *x*_1_ + 5.3 *x*_2_ + 0.863	0.746 **	24
U = 14.6	y = 5.092 *x*_1_ + 5.507 *x*_2_ + 2.332	0.785 **	24
U = 18.5	y = 5.375 *x*_1_ + 9.392 *x*_3_ − 1.657	0.819 **	24
U = 22.4	y = 4.485 *x*_1_ + 8.748 *x*_3_ + 0.276	0.763 **	24
U = 26.2	y = 4.993 *x*_1_ + 7.177 *x*_3_ + 2.749	0.755 **	21
U = 30.1	y = 6.263 *x*_1_ + 9.987	0.485 **	19
U = 33.9	y = 4.482 *x*_2_ + 8.756	0.349 *	14
U = 37.8	-	-	6

Note: U represents wind speed, y represents torque, *x*_1_ represents leaf area, *x*_2_ represents plant fresh weight, *x*_3_ represents the height of the center of gravity, and n represents the sample size, - represents that there is no significant variable, * indicates significance at 0.05, ** indicates significance at 0.01.

## Data Availability

The original contributions presented in this study are included in the article. Further inquiries can be directed to the corresponding author.

## Abbreviations

The following abbreviation is used in this manuscript:

U	Wind speed
